# Holistic Therapeutic Approaches Improve Functional Mobility in Patients With Postoperative Vertebral Compression Fracture (VCF): A Case Report

**DOI:** 10.7759/cureus.44032

**Published:** 2023-08-24

**Authors:** Arjavi A Pakhan, Manali A Boob, Kamya J Somaiya, Pratik Phansopkar

**Affiliations:** 1 Physiotherapy, Ravi Nair Physiotherapy College, Datta Meghe Institute of Higher Education & Research (DU), Wardha, IND; 2 Musculoskeletal Physiotherapy, Ravi Nair Physiotherapy College, Datta Meghe Institute of Higher Education & Research (DU), Wardha, IND; 3 R&D, RNPC, Ravi Nair Physiotherapy College, Datta Meghe Institute of Higher Education & Research (DU), Wardha, IND

**Keywords:** schobers test, rehabilitation, pain management, pedicel screw fixation, lumbar fracture

## Abstract

For an unusual spinal injury that frequently results in a traumatic fracture of the lumbar spine, surgical procedures, including spinal fixation and osteotomies, are commonly needed for the therapy of complicated spinal pathologies to regain stability and relieve pain. A 55-year-old man complained of lower back pain while lifting heavy objects at work. He was taken to Acharya Vinoba Bhave Rural Hospital (AVBRH), where a radiological examination revealed a fracture of the L3 vertebra. He underwent surgery for the fracture, which included an L2-L4 spinal fixation operation and an L3-level osteotomy. The patient was sent to the physiotherapy department for postoperative recovery after the surgery. The rehabilitation program was designed according to the patient's condition. The goals were maximizing functional recovery, better pain management, and improving the participant's health and quality of life. The novel rehabilitation strategy strongly emphasized a multifunctional, patient-centric approach and evidence-based methodologies. The goals of the therapy were to regain full range of motion, gradually increase axial loading, and keep the supporting muscles strong.

## Introduction

The lower back is mainly composed of lumbar vertebrae. They originate at the thoracolumbar junction, or the beginning of the lumbar curve, and extend to the sacrum [1]. In the context of trauma, bone fragility, sepsis, tumors, or other bone disorders, vertebral fractures are caused by incorrect axial compression regardless of whether there is a rotational component, distraction, or dislocation [2]. Fractures of the spinal column develop due to a bone's biomechanical failure following an axial or compressive force. This fracture puts the front column of the spine at risk, which also puts the front half of the vertebral body and the anterior longitudinal ligament at risk [1]. The result is the recognizable wedge-shaped malformation. These fractures exclude the posterior half of the vertebral body, the posterior osseous elements, and the posterior ligamentous complex [3]. Vertebral compression fracture (VCF) is the most prevalent fracture [4]. However, the distribution of compression fractures shows a bimodal pattern, with younger individuals suffering from these wounds due to high-energy mechanics [5]. According to recent research, 60%-75% of VCF occur in the thoracic and lumbar spine, and another 30% happen in the L2-L5 area. About 50% of spine fractures in younger patients are caused by car accidents, with falls accounting for another 25% of cases [6]. Back discomfort and a fracture shown on radiography, most frequently between T8 and L4, might be a patient's symptoms. Individuals with an acute fracture could have discomfort that starts suddenly and worsens with movement, coughing, sneezing, as well as lifting. While kyphosis and discomfort along the midline of the spine might be seen, physical examination results are frequently typical. In addition to kyphosis, chronic VCF can also cause a height decrease. Consequences might include spinal cord compression, venous thromboembolism, bone loss, muscular weakness, pressure sores, and weakened bones [7]. The fracture's nature and any neurologic damage will primarily determine the surgical alternatives. The most frequent surgical options for these individuals are vertebroplasty or kyphoplasty, which involves cement augmentation. Once the vertebral height has been restored, cement is then injected [8]. We described a case of an L3 VCF that was treated with an osteotomy at the L3 level and a spinal fixation operation at L2-L4. A vertebral fracture can significantly affect a person's condition. There is growing evidence that physical therapy, which uses manual methods and exercise prescription, may play a significant role in treatment.

## Case presentation

**Figure 1:**
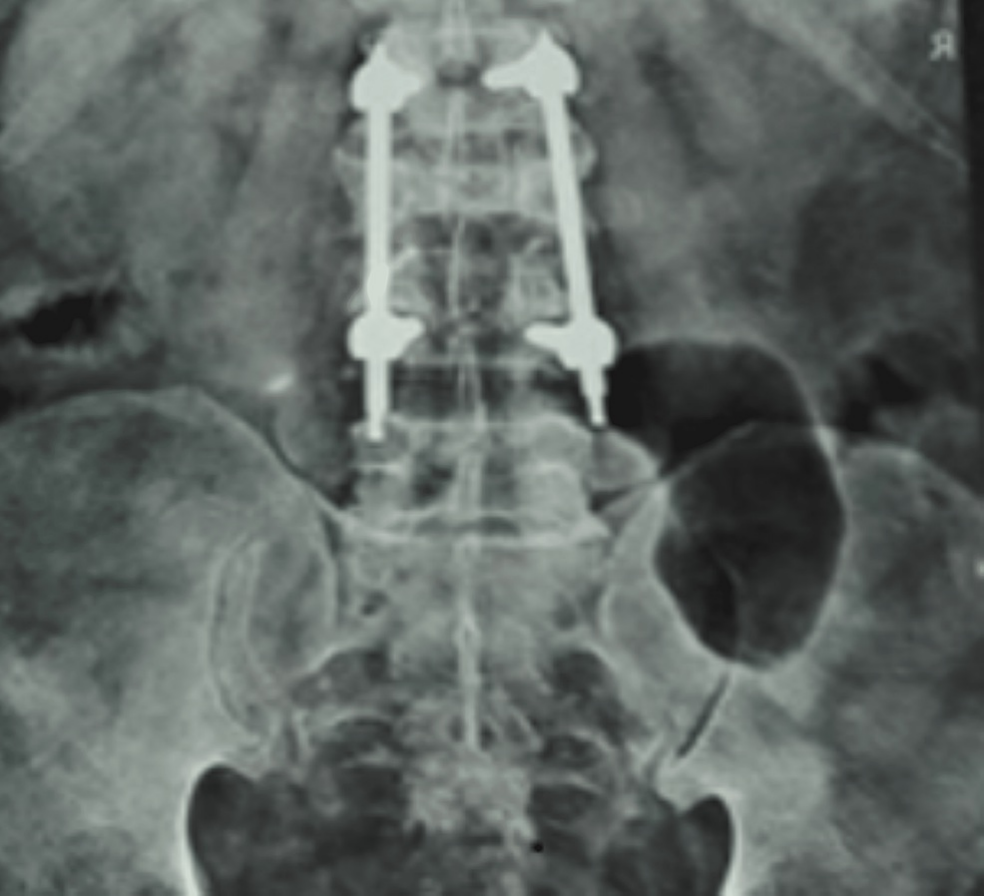
Post-operative radiological image of lumbar spine with pedicle screws and connecting rods fixation at L2-L4 lumbar level. L, lumbar

A 55-year-old male, while lifting heavy weights at the workplace, felt a sudden pull and pain over his back. By the evening, there was increased pain over the lower back region with difficulty in walking and bed mobility activities. There was dull aching and shooting type of pain present in his back area, he visited Acharya Vinoba Bhave Rural Hospital (AVBRH) with the same complaint. A radiological investigation was done, which shows an L3 compression fracture with a neuro deficit. A spinal fixation procedure at L2-L4 with an osteotomy at the L3 level is shown in X-ray (Figure 1). After surgery, the patient's overall status was normal. There were no complications during or after the surgery. He was recommended for physiotherapy for further management. The patient was examined in a supine lying position. The pelvis was neutral, both legs were extended, and the ankles were in mild plantar flexion. It was observed that swelling was present in the lumbar region. A rise in the local temperature was felt upon palpation. Grade two tenderness was present along the suture at the lumbar area. A paraspinal muscle spasm was present. The patient has a bilateral restricted range of hip joints and the lumbar spine.

Timeline

The patient was admitted to the hospital on the 3rd of March 2023, diagnosed with a lumbar fracture diagnosed on the 3rd of March 2023, and underwent surgery on the 5th of March 2023. Physiotherapy began on the 6th of March, 2023. The suture was removed on the 16th of March, 2023. After discharge from the hospital, patient physiotherapy was continued for three weeks.

Intervention

The patient and their family members should receive education regarding the disease and advice on good posture to prevent further fracture displacement or spinal cord injuries. Short-term goals for the post-operative period included reducing respiratory issues, discomfort, and edema, improving joint mobility, increasing joint power, and promoting support for independent daily living activities. In the long run, we wanted to lessen pain and edema, increase joint mobility and strength, and improve walking, balancing, and proprioception-free aerial activities. Here is a week-by-week recovery plan as mentioned below as shown in Table 1.

**Table 1:** Phases of rehabilitation.

Phases of rehabilitation	Physiotherapeutic goals	Physiotherapeutic intervention
Phase 1 (0-1 days)	Patient education and to initiate bed mobility	Patient education, pain management, and deep breathing exercises to prevent respiratory complications. Lower limb movements and log rolling to avoid strain on the spine. Ankle-toe movements and relaxation techniques.
Phase 1 (2-3 days)	To promote circulation, muscle atrophy, and to maintain range of motion	Ankle pumps, static exercises, active-assisted motions, ankle-toe movement, heel slides, and straight leg raises. Bedside sitting with a brace on.
Phase 1 (4-7 days)	To initiate strength training and mobility	Static back and static abdomen exercises to strengthen the core muscles. Bedside standing with a brace on and using a walker.
Phase 2 (weeks 1-3)	To increase the strength of the muscle, to improve balance, and educate the patient for git training	Continuation of all the exercises of phase one, including static back and static abdomen. Introduction of sit-to-stand routines and active motions In week two focus on core strengthening exercises and gait training with the use of a walker. Task-oriented exercises for balance improvement were started. In week three gradual reduction in the use of a walker during gait training. Increase in the difficulty level of strengthening exercises like dynamic squats, lunges, and mini squats.
Phase 3 (weeks 4-8)	To improve balance, mobility, strength, and endurance. Incorporating more dynamic exercises to challenge and enhance overall function.	Continuation of phase two exercises, with an emphasis on gait training without a walker. Initiation of hurdle walking, tandem walking, marching in place, high stepping, and stair climbing to enhance balance and mobility. Focus on further progression of strengthening exercises, including dynamic squats, lunges, and mini squats. Incorporation of home exercises for a consistent strengthening routine.

*Phase 1*: Inpatient phase [0-7 days]: Focuses on patient education, pain relief, back muscular strength maintenance, and ergonomic guidance. The illness was communicated to the patient, in addition to the do’s and don'ts. The patient received instructions on proper lifting techniques. Lower limb movements, log rolling, upper limb movements, and bed mobility were the primary focus. The emphasis was placed on supported sitting, bedside sitting with a brace on, and bedside standing with a brace on and a walker. To lessen discomfort, retrain the lungs, and prevent post-operative edema, cryotherapy, relaxation techniques, correct posture, deep breathing exercises, and ankle-toe movements were administered. He was given actively aided motions that proceeded to active lower limb movements such as ankle-toe movement, heel slides, straight leg raise, abduction, and adduction. To avoid bad spine motions, log rolling was taught. Around two to three days following surgery, bedside sitting with a brace was performed. After about a week of recovery from surgery, bedside standing with a brace on and a walker was encouraged. To improve the strength of the core muscles, we started with a static back and a static abdomen.

*Phase 2*: Out-patient phase [1-3 weeks]: Most of the workouts from Phase 1 were continued in Phase 2, along with activities like walking with a brace on and core strengthening exercises being the major emphasis. To facilitate core strengthening, sit-to-stand routines, active motions, and statics were all still being done. Active pelvic bridging was used to improve core activation while wearing a brace and using a walker to stimulate the development of stronger lower limbs.

**Figure 2:**
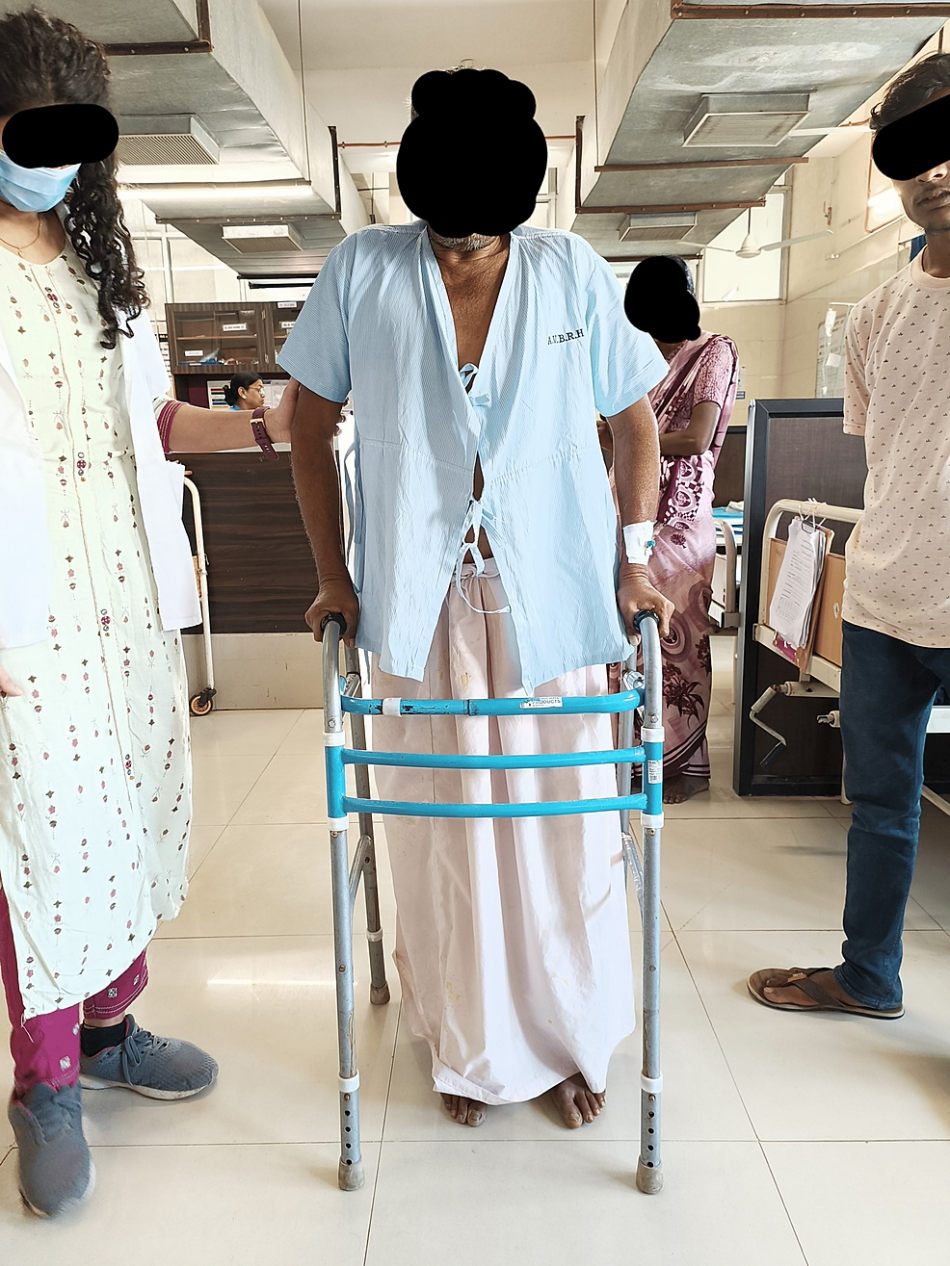
Walking with frame assistance.

*Phase 3*: Out-patient [4-8 weeks]: During this period, balance, mobility, strength, and endurance need to be encouraged. The entirety of the Phase 2 workouts was continued, with an accent on strengthening and gait training. Phase 2 activities were progressed to without the use of a walker, including walking sit-to-stand and weighted reach-outs for balance. Gait training (Figure 2) was facilitated by hurdle walking, tandem walking, marching in place high stepping, and stair climbing. Strengthening exercises featured dynamic squats, lunges, and mini squats. These exercises were also being done at home as part of a strengthening regimen.

The purpose of this surgery is to support and stabilize the damaged spinal segments, relieve discomfort, and encourage vertebral fusion for long-term healing. To speed up recovery and restore function after surgery, a well-planned rehabilitation program is necessary.

Outcome measure

Pre- and post-outcome measure after the physiotherapeutic rehabilitation is mentioned in Tables 2-4.

**Table 2:** Range of motion.

Joint movement	Pre-treatment		Post-treatment	
Side	Right	Left	Right	Left
Hip flexion	0-40 °	0-50 °	0-100°	0-120°
Hip extension	0-15°	0-20°	0-30°	0-30°
Hip abduction	0-30°	0-30°	0-30°	0-30°
Hip adduction	0-20°	0-20°	0-20°	0-20°
Hip internal rotation	0-30°	0-35°	0-40°	0-40°
Hip external rotation	0-40°	0-40°	0-50°	0-50°
Knee flexion	0-60°	0-60°	0-130°	0-135°
Knee extension	60-0°	60-0°	130-0°	135-0°
Ankle plantarflexion	0-30°	0-30°	0-30°	0-40°
Ankle dorsiflexion	0-05°	0-10°	0-20°	0-20°

**Table 3:** Manual muscle testing.

Muscles	Pre-treatment		Post-treatment	
Side	Right	Left	Right	Left
Hip flexors	3/5	3/5	4/5	4/5
Hip extensors	3/5	3/5	4/5	4/5
Hip adductor	3/5	3/5	4/5	4/5
Hip abductors	3/5	3/5	4/5	4/5
Hip internal rotators	3/5	3/5	4/5	4/5
Hip external rotators	3/5	3/5	4/5	4/5
Knee flexors	3/5	3/5	4/5	4/5
Knee extensors	3/5	3/5	4/5	4/5
Ankle plantarflexors	4/5	4/5	5/5	5/5
Ankle dorsiflexors	4/5	4/5	5/5	5/5

**Table 4:** The outcome measure.

Parameters	Pre-operative	Post-operative
Lumbar flexion	0-20	0-50
Lumbar extension	05	15
Lumbar (Schober’s test)	The difference is 3 cm	The difference is 6 cm
Isometric extensor test	Trace (merely a slight muscle activation with no mobility)	Fair (extend the lumbar spine and elevate the sternum off the bed/floor with your hands at your sides)
Isometric abdominal grading	Poor (able to elevate the upper body till the scapula rises off the table with arms outstretched towards the knee)	Good (able to elevate the upper trunk till the scapula clears the table with folded arms on the chest)
Oswestry disability index	70% impairment	30% impairment

## Discussion

This case describes the osteotomy at the L3 level and spinal fixation at the L2-L4 level; the patient's condition rehabilitation goals were developed, starting with low-intensity normal range of motion exercises and progressing to high-intensity and resisted exercises which helped him to perform all activities of daily living [9]. In the short and long terms, physical therapy should help with early mobilization during the acute phase and injury prevention. Forward flexion should be avoided in the early phase -- this precaution should be taken. Hence, the exercises recommended should serve two purposes: improving posture and ambulation while reducing the risk of future falls and strengthening the patient's supporting axial musculature, particularly the spine extensors [10]. There should be many different applications for manual therapy to improve functional abilities and reduce back pain. Interferential treatment is a method that creates a medium-frequency current by combining two low-frequency currents; this approach also aids in pain relief [11]. Interferential treatment is a method that combines two low-frequency currents to generate a medium-frequency current that also helps to alleviate pain. Hydrocollateral packs are used for superficial heating, which relaxes the muscles and promotes blood circulation [12]. According to McCarthy et al., early mobility should be announced as early as tolerated. If pain is unbearable, bed rest may be advised as the first course of treatment; however, this might result in muscle and bone weakness, bedsores, and deep vein thrombosis. Inconclusive data was discovered by the American Academy of Orthopedic Surgeons (AAOS) about the advantages of bed rest in treating VCF [7]. Individuals with VCFs and osteoporosis will likely benefit from physical therapy. Fewer studies have shown that home exercise regimens can reduce pain and enhance balance and quality of life [13]. Strengthening the back extensors can increase strength and bone density while lowering the risk of future VCF. All people with osteoporosis benefit from exercise [14]. A four-week program decreased fall risk and patient anxiety about falls, enhanced physical activity level, and reduced backache and strength. Using digital analysis, it was also demonstrated that the patient's posture and gait had improved [15]. It is important to remember that the patient should not be alarmed. Additionally, the patient should not be anxious while performing activities [16].

## Conclusions

In this case report, a 55-year-old male underwent an L3 osteotomy and L2-L4 spinal fixation procedure. With the aid of routine therapy, he was able to perform activities of daily living independently. Overall, the success of this case highlights the value of customized rehabilitation strategies in post-surgical recovery. The medical team was able to construct a customized program that encouraged healing, strength, and mobility, thereby increasing the patient's quality of life, by taking into account the patient's particular condition, needs, and objectives. The joint effort of medical personnel and the patient was critical in attaining the intended results, emphasizing the need for tailored treatment in comparable circumstances.
